# Symbiotic lifestyle triggers drastic changes in the gene expression of the algal endosymbiont *Breviolum minutum* (Symbiodiniaceae)

**DOI:** 10.1002/ece3.5910

**Published:** 2019-12-12

**Authors:** Keren Maor‐Landaw, Madeleine J. H. van Oppen, Geoffrey I. McFadden

**Affiliations:** ^1^ School of BioSciences The University of Melbourne Melbourne Vic. Australia; ^2^ Australian Institute of Marine Science Townsville Qld Australia

**Keywords:** *Breviolum minutum*, *Exaiptasia diaphana*, free‐living, *in hospite*, symbiosis, transporters

## Abstract

Coral–dinoflagellate symbiosis underpins the evolutionary success of corals reefs. Successful exchange of molecules between the cnidarian host and the Symbiodiniaceae algae enables the mutualistic partnership. The algae translocate photosynthate to their host in exchange for nutrients and shelter. The photosynthate must traverse multiple membranes, most likely facilitated by transporters. Here, we compared gene expression profiles of cultured, free‐living *Breviolum minutum* with those of the homologous symbionts freshly isolated from the sea anemone *Exaiptasia diaphana*, a widely used model for coral hosts. Additionally, we assessed expression levels of a list of candidate host transporters of interest in anemones with and without symbionts. Our transcriptome analyses highlight the distinctive nature of the two algal life stages, with many gene expression level changes correlating to the different morphologies, cell cycles, and metabolisms adopted *in hospite* versus free‐living. Morphogenesis‐related genes that likely underpin the metamorphosis process observed when symbionts enter a host cell were up‐regulated. Conversely, many down‐regulated genes appear to be indicative of the protective and confined nature of the symbiosome. Our results emphasize the significance of transmembrane transport to the symbiosis, and in particular of ammonium and sugar transport. Further, we pinpoint and characterize candidate transporters—predicted to be localized variously to the algal plasma membrane, the host plasma membrane, and the symbiosome membrane—that likely serve pivotal roles in the interchange of material during symbiosis. Our study provides new insights that expand our understanding of the molecular exchanges that underpin the cnidarian–algal symbiotic relationship.

## Graphical abstract

The symbiosis between corals and dinoflagellate algae underpins the evolutionary success of corals reefs. Our study focuses on algal symbiont gene expression and targets differentially expressed putative transporters that might serve pivotal roles in the interchange of material during symbiosis. Our study emphasizes the great significance of transmembrane transport to the success of the symbiotic relationship.

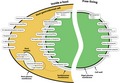

## 1 INTRODUCTION

Symbiosis is a powerful evolutionary strategy that combines skill sets from separate species to meet environmental challenges (Antonelli, Rutz, Sammarco, & Strychar, 2016). Dinoflagellate symbionts of corals are a classic example of such a partnership. Symbiotic dinoflagellates originated approximately 160 mya (LaJeunesse et al., 2018) and diversified together with their coral hosts during the middle‐to‐late Jurassic (~176–161 mya (Stanley & Swart, 1995). Symbiotic dinoflagellates reside within the inner tissue layer of the host, the gastrodermis (Davy, Allemand, & Weis, 2012).

In most scleractinian coral species, each new generation has to acquire the algal symbionts from the environment (Baird, Guest, & Willis, 2009). Algae enter the mouth of the gastric cavity and are then taken up into larval gastrodermal cells by a phagocytosis‐like process (Schwarz, Krupp, & Weis, 1999). Symbionts apparently avoid digestion, and the phagosome converts to a “symbiosome” (Davy et al., 2012; Mohamed et al., 2016), which is protected from host lysosomal degradation and controls nutrient flux between the partners (Davy et al., 2012; Fitt & Trench, 1983).

Successful exchange of molecules through membranes is a key feature enabling mutualistic partnerships. Symbiodiniaceae algae (formerly *Symbiodinium*; LaJeunesse et al., 2018) translocate photosynthate into their host, fulfilling a large component of its nutritional requirements. In return, the algae derive benefits from the host in the form of nutrients and shelter from predation (Muscatine & Porter, 1977). Glucose is the major primary carbon metabolite translocated from the symbiont to the host (Burriesci, Raab, & Pringle, 2012), while the host supplies nitrogen to the symbiont in the form of ammonium (Kopp et al., 2013; Pernice et al., 2012; Sproles et al., 2018).

Compounds exchanged between the symbiont and the host need to traverse at least three membranes: the algal plasma membrane, the symbiosome membrane, and the host cell membrane. Furthermore, the symbiosome barrier comprises two membrane components: a single outer membrane believed to be derived from the host phagosome and a multilayered inner membrane derived from the symbiont (Wakefield, Farmer, & Kempf, 2000; Wakefield & Kempf, 2001). All these membranes form a boundary between the symbiont and the host: All cell‐to‐cell communications, as well as transport of gasses and translocation of photosynthesis products, must traverse this interface (Wakefield et al., 2000).

The exchange of compounds between the host and the symbiont is likely facilitated by transporters, probably within each of the membranes (Rands, Loughman, & Douglas, 1993). ATPase activity on both the Symbiodiniaceae plasma membrane and the symbiosome of *Anemonia viridis* (Rands et al., 1993) supports the notion of active membrane transport, allowing the host and the symbiont to control the flux of nutrients. Thus far, just a single ABC transporter protein driven by ATP hydrolysis has been identified in symbiosomes isolated from the symbiotic anemone *Exaiptasia diaphana* (Peng et al., 2010; formerly *pulchella*; Grajales & Rodríguez, 2014), and therefore, our understanding of the membrane gateways between symbiont and host is in its infancy.

Recent comparative analyses of genome, transcriptome, and protein sets showed that the family Symbiodiniaceae is enriched with domains involved in transmembrane transport when compared to other eukaryotes (Aranda et al., 2016) or even other dinoflagellates (Liu et al., 2018). Domains of bicarbonate transporters, carbonic anhydrase, and ammonium transporters are particularly enriched when compared to other dinoflagellates (Aranda et al., 2016), which suggests that expansion of transporters has occurred in Symbiodiniaceae as an adaptation to symbiotic lifestyle (Aranda et al., 2016). Previous bioinformatic/phylogenetic studies have identified numerous transporter genes in Symbiodiniaceae (Aranda et al., 2016; González‐Pech, Ragan, & Chan, 2017; Liu et al., 2018; Sproles et al., 2018), but their roles in symbiosis are yet to be determined. Although the mutualistic symbiosis between corals and Symbiodiniaceae has been investigated since the late 1800s, the literature still has huge gaps in understanding how metabolic trafficking is taking place and which membrane transporters assist in the process.

Symbiodiniaceae algae adopt highly divergent morphologies, cell cycles, and life histories depending on whether they exist *in hospite* or free‐living in liquid culture (Trench, 1993). For instance, in log‐phase liquid culture Symbiodiniaceae cells are mostly motile (mastigote) with two flagella and the characteristic gymnodinioid shape. Conversely, inside the host where Symbiodiniaceae are confined by the symbiosome, the cells are coccoid, lack flagella (Trench, 1993), are larger (Pasaribu et al., 2015), have relatively smaller plastids (Pasaribu et al., 2015), and have thinner walls (Palincsar, Jones, & Palincsar, 1988; Pasaribu et al., 2015; Wakefield et al., 2000). *In hospite*, Symbiodiniaceae grow 1–2 orders of magnitude slower than in culture (Stambler, 2011; Wilkerson, Kobayashi, & Muscatine, 1988), and photosynthesis rates are also depressed (Bhagooli & Hidaka, 2003; Chen, Yeh, Wang, Li, & Chen, 2012; Deane & O'Brien, 1978). Finally, *in hospite* Symbiodiniaceae have different lipid profiles to those of cultured Symbiodiniaceae (Chen et al., 2015). Clearly, major morphological, physiological, and biogenesis changes accompany the switch from free‐living to symbiont lifestyle when Symbiodiniaceae algae enter into symbiosis with a host.

Here, we identify a set of transporter genes that are likely involved in the nutrient exchange between Symbiodiniaceae endosymbionts and their cnidarian host. To identify host transporters involved in host–symbiont nutrient exchange, we searched the literature for *E. diaphana* transporters that are differentially expressed in aposymbiotic versus symbiotic animals (Lehnert et al., 2014; Oakley et al., 2016; Wolfowicz et al., 2016) and analyzed these candidate transporters of interest in our system of symbiotic and aposymbiotic *E. diaphana* sea anemones using real‐time PCR. For the exploration of algal symbiont transporters, we conducted a comparison of gene expression profiles from RNAseq data between free‐living cultured *Breviolum minutum* (ITS2 type B1) and symbiotic algae of the same strain that had been freshly isolated from the sea anemone, *Exaiptasia diaphana*, of Great Barrier Reef origin. We examined this data set for genes that are differentially expressed between the two life stages. In addition to transporters, we aimed to generate a gene expression compendium providing a molecular window into the dramatic morphological, physiological, and life‐history differences for Symbiodiniaceae *in hospite* or free‐living states.

## 2 MATERIAL AND METHODS

### 2.1 Symbiotic anemone cultures

Three genotypes (AIMS2, AIMS3, and AIMS4; as characterized by genome‐wide SNP analysis, Dungan et al., 2019) of *Exaiptasia diaphana* sea anemones were collected from the National Sea Simulator at the Australian Institute of Marine Science (AIMS, Australia). These animals are of Great Barrier Reef (GBR) origin (most likely from the central GBR), and all were found to host the same Symbiodiniaceae strain (Dungan et al., 2019). Three replicate tanks with clonal *E. diaphana* were kept on three different shelves of a walk‐in constant temperature room under the same conditions: constant temperature of 26°C, 12:12 light:dark photoperiod cycle, and 15 μmol photons m^−2^s^−1^ (the GBR anemones are highly light sensitive and they thrive under low light conditions; Dungan et al., 2019), along with constant air supply. Anemones were maintained in 2.4‐L plastic containers with sea water (Red Sea Salt, salinity of 34 ppt). Anemones were fed ad libitum twice a week with freshly hatched *Artemia* sp. nauplii (hatched overnight under constant air supply), and once a week, filamentous algae were removed immediately prior to a full water change.

### 2.2 Preparation of aposymbiotic anemones

Aposymbiotic anemones of the three genotypes were generated by chemical bleaching using a menthol‐diuron treatment, as described by Matthews et al. (2015). The procedure was repeated for four consecutive days per week and lasted a total of 6 weeks. The anemones were kept in a constant temperature room under the same conditions as described above. Samples were taken for RNA extraction after the 6 weeks, and complete bleaching was verified by the absence of algal chlorophyll auto‐fluorescence with a Leica M205 FA dissecting microscope with a Leica DFC450 C camera, using GFP LP (blue) filter.

### 2.3 Symbiont cultures

*Breviolum minutum* cells were isolated and cultured from a single‐genotyped *E. diaphana* anemone (Tortorelli et al. in press). Three replicate subcultures were generated in cell culture flasks (0.2‐μm membrane vented cap) with 0.2 μm filtered seawater (FSW) supplemented with 1× Diago's IMK medium (Novachem). Cultures were kept in a growth chamber (740FHC LED, HiPoint) under a constant temperature of 26°C, 12:12 light:dark photoperiod cycle, and 60 μmol m^−2^S^−1^ photons. Cultures were routinely monitored by light microscopy to assess their health (cells are intact, vital, and motile cells are present). The replicate subcultures were kept and maintained for at least a month prior to RNA extractions. Prior to RNA extraction, cells were counted using an automated cell counter (countess™ II FL automated cell counter with EVOS™ GFP light cube for detection of fluorescence).

### 2.4 RNA extraction

RNA was extracted from the two life stages of *B. minutum*, cultured, and freshly isolated, and from symbiotic and aposymbiotic *E. diaphana* anemones.

Cultured symbionts: ~1 × 10^7^ cells from each of the three replicate flasks were processed for RNA extraction. RNA was extracted according to Rosic and Hoegh‐Guldberg (2010). Briefly, 8 ml of culture was centrifuged at 5,450 g for 15 min, the supernatant was removed, and the pellet was resuspended in 1× Diago's IMK medium. A second round of centrifugation of 10 min at 10,000 g resulted in a pellet that was resuspended in TRIzol (Invitrogen Thermo Fisher Scientific). Cells were homogenized using 710–1,180 mm acid‐washed glass beads (Sigma) and a TissueLyser II (Qiagen) for 90 s at 30 Hz. The upper aqueous phase containing RNA was retrieved following incubation with chloroform (Sigma‐Aldrich) and centrifugation of 15 min at 12,000 g. The aqueous phase was mixed with 0.5 volume of absolute ethanol and transferred to a spin column of the RNAeasy plant RNA extraction kit (Qiagen) for RNA precipitation and washes, according to manufacturer instructions.

Freshly isolated symbionts: Algal symbionts were isolated from a pool of 10–12 clonal (of the same genotype: AIMS4) symbiotic *E. diaphana* anemones from the three replicate tanks. Anemones were homogenized using a sterile glass homogenizer, and symbiont cells were pelleted by centrifuge at 5,000 g for 5 min. The supernatant containing the host cells was discarded, and the symbiont cells were suspended with FSW. To minimize host contamination in RNA extracts, this step was repeated four times until the supernatant was clear. The rest of the RNA extraction protocol was the same as described above for the cultured symbionts.

Symbiotic and aposymbiotic anemones: Three replicates of each, representing three distinct genotypes (AIMS2–4), were taken for RNA extractions. RNA was extracted using TRIzol (Invitrogen Thermo Fisher Scientific) following the manufacturer's instructions with a few adjustments: Frozen anemones were homogenized in cold TRIzol (kept at 4°C) using glass homogenizer and, following centrifugation, were further homogenized by 425–600 mm acid‐washed glass beads (Sigma) using a TissueLyser II (Qiagen) for 30 s at 30 Hz. RNA was precipitated in 250 μl ice‐cold isopropanol and 250 μl 0.8 M sodium citrate in 1.2 M sodium chloride.

The concentration of the RNA was measured using a DeNovis DS‐11 spectrophotometer, and the RNA integrity was assessed by agarose gel electrophoresis and evaluated based on clear 28S and 18S ribosomal RNA bands. *B. minutum* RNA quality and quantity were further assessed by the Australian Genome Facility (AGRF) using the Agilent 2100 Bioanalyzer system with a RNA 6000 Nano kit.

### 2.5 Real‐time PCR for anemone candidate transporters of interest

Real‐time polymerase chain reaction (qPCR) analysis was performed for 14 candidate transporters of interest that were found to be significantly down‐regulated in anemones without endosymbionts comparing to symbiotic anemones in previous transcriptome/proteomic papers (Lehnert et al., 2014; Oakley et al., 2016; Wolfowicz et al., 2016; Table S1). Complementary DNAs were synthesized from 0.25 μg of total RNA using RevertAid First Strand cDNA Synthesis Kit (Thermo Scientific), according to manufacturer's instructions. Specific qPCR primers were designed to amplify a 100–200 bp PCR product (Table S2). Amplicon identity was verified using Sanger sequencing (AGRF). cDNA aliquots were diluted 1:5, and a 2 μl sample was used in technical triplicates for 10 μl qPCR reactions including 5 μl of SYBR FAST Universal Kit qPCR Master Mix (KAPA Biosystems) and 0.2 μl each of forward and reverse primer, for 40 cycles. A melt curve obtained by incubating the reactions for 10 s at 0.5°C increments between 60°C and 90°C was generated for each amplicon, to test for nonspecific amplification. The comparative ΔΔ*CT*s method was used, and fold changes were calculated using the 2^−ΔΔ^ *^Ct^* formula to estimate the relative difference in the number of transcripts between the aposymbiotic and symbiotic samples. Four potential house‐keeping genes were tested: 28S ribosomal RNA gene (Rodriguez‐Lanetty, Phillips, & Weis, 2006), beta‐actin (Rodriguez‐Lanetty et al., 2006), 40S ribosomal protein S7 (Lehnert et al., 2014), and encoding ribosomal protein L11 (RPL11; Sorek et al., 2018). Samples were normalized to the latter, which was the best performing one and showed the most stable expression pattern across all samples. In order to distinguish statistically significant results, we utilized an independent samples test for each gene (2‐tail *p* < .05) using the SPSS software (Version 20.0., IBM Corp).

### 2.6 Transcriptome assembly

Total RNA (400 ng) of cultured and freshly isolated *B. minutum* (three biological replicates of each) was used for Illumina TruSeq Stranded mRNA library prep kit, with polyA purification, and sequenced on the Illumina HiSeq platform (paired‐end, 125 bp, ~34–44 × 10^6^ reads per sample) by AGRF. The Illumina bcl2fastq 2.20.0.422 pipeline was used to generate the sequence data. The data generated met the AGRF quality standards. The Fastq files were deposited at SRA database under the accession number PRJNA544863.

RNAseq analysis was conducted using the Galaxy interface, a web‐based platform for data‐intensive biological research (Afgan et al., 2015). Quality of raw reads was assessed via FastQC (https://www.bioinformatics.babraham.ac.uk/projects/fastqc/; Andrews, 2010). Trim Galore software (http://www.bioinformatics.babraham.ac.uk/projects/trim_galore/) was used for trimming sequencing adapters and low‐quality reads. In order to minimize host and bacterial contamination in the assembled transcriptome, the reads were searched against a custom‐made database comprised of publicly available Symbiodiniaceae and cnidarian transcriptomes in order to maximize the sequences coverage (including *B. minutum* [Shoguchi et al., 2013], *Symbiodinium microadriaticum* [Aranda et al., 2016], *E. diaphana* [Baumgarten et al., 2015], and *Stylophora pistillata* [Voolstra et al., 2017]), using BLASTN. Only reads for which their best matching annotation was significantly (*e*‐value < 5 × 10^−5^) mapped to an endosymbiotic alga were further used for the assembly.

Average library size was 6 million and 30 million assigned reads for the freshly isolated and cultured samples, respectively. De novo assembly was conducted in Trinity (Grabherr et al., 2013; Galaxy Version 2.4.0.0) using the default parameters, resulting in 75,383 assembled contigs. Putative coding regions were extracted from the transcriptome assemblies using the TransDecoder software (Haas et al., 2013) providing all the CDS and proteins from the assembly and retaining the longest representative open reading frame per transcript, and resulted in 58,619 contigs. The quality of the assembled transcriptome was assessed by mapping the filtered reads back to the assembled transcriptome using Bowtie 2 (Langmead & Salzberg, 2013) and achieving ~97% alignment rate per sample. Additionally, contig Ex90N50, Ex90 transcript count (Grabherr et al., 2011), and %GC were computed using QUAST (Gurevich, Saveliev, Vyahhi, & Tesler, 2013). The completeness of the transcriptome was assessed using BUSCO (Simão, Waterhouse, Ioannidis, Kriventseva, & Zdobnov, 2015) and estimated at 78.5% (see (Levin et al., 2016) for a review of completeness of other Symbiodiniaceae transcriptomes).

### 2.7 Differential gene expression analysis

Transcript quantification was generated via Salmon (Patro, Duggal, Love, Irizarry, & Kingsford, 2017) as filtered trimmed reads were back aligned to the assembled transcriptome. Annotations were created by blasting the translated transcriptome against the UniProtKB (Swiss‐Prot) database by BLASTP (Camacho et al., 2009; Cock, Chilton, Grüning, Johnson, & Soranzo, 2015). Annotations were also created from the nr database using Diamond (version 0.9.18) in sensitive mode (Buchfink, Xie, & Huson, 2015). Filtering was applied to the BLASTP UniProtKB results in order to increase the certainty of obtaining true homologs. Filtering parameters were set at an e‐value threshold of 5.10^−5^, >20% alignment identity, and >50% query coverage. Following filtration, 16,484 annotated transcripts were left. Degust tool (https://drpowell.github.io/degust/) with EdgeR method was used to achieve statistics, to compute normalized log2(FC) (*in hospite* vs. cultured) to the transcripts and to draw a multidimensional scaling (MDS) plot. A hierarchical clustering heat map was generated using TPM values (transcript per million) and based on Euclidian distances in Excel XLSTAT.

Differentially expressed genes (DEGs) were defined as those with an adjusted *p*‐value < .05. Functional gene enrichment analysis was done, using the UniProtKB annotations in David Bioinformatics Resources (Huang, Sherman, & Lempicki, 2009), and enriched Gene Ontologies (GO; biological process, cellular component, molecular function, KEGG pathways, INTERPRO domain) were retrieved.

Some GO categories were grouped into “groups‐of‐interest” according to a functional annotation clustering analysis conducted in David Bioinformatics Resources (Huang et al., 2009) and ancestors' terms retrieval in CateGOrizer (Zhi‐Liang, Bao, & James, 2008). An enrichment score was calculated for each group of GOs, which was defined as the minus log of the geometric mean of the all the *p*‐values of the GO categories within the group (Huang et al., 2009).

Annotations were filtered by “transporter”/“solute carrier” as a keyword, creating a list of potential transporters that are differentially expressed. We have generated another data set of potential sugar transporters, by searching the annotations and GOs with keywords.

Symbiont differentially expressed transporters transcripts and also anemone candidate transporters of interest were further analyzed by protein subcellular localization prediction tools; protein sequences were run through several bioinformatic prediction tools available online (Almagro Armenteros, Sønderby, Sønderby, Nielsen, & Winther, 2017; Chou & Shen, 2010; Chou, Wu, & Xiao, 2010; Emanuelsson, Nielsen, Brunak, & von Heijne, 2000; Lin, Fang, Xiao, & Chou, 2013; Pierleoni et al., 2011). The best performing one, DeepLoc (Almagro Armenteros et al., 2017), was selected by testing prediction output of proteins that their localization in the cell was experimentally tested (such as: Dani, Ganot, Priouzeau, Furla, & Sabourault, 2014). DeepLoc prediction algorithm relies only on sequence information and was demonstrated to achieve a good accuracy (Almagro Armenteros et al., 2017). Only proteins that were predicted to be membranal and not soluble and that were ranked with high a hierarchical tree likelihood (>0.5), were taken into consideration.

In an attempt to further predict which of the host's candidate transporters of interest might be localized to the symbiosome membrane, we focused on those that were predicted to the lysosome/vacuole. We used host's transcripts that were predicted to the lysosome/vacuole, to search the genome of the nonsymbiotic anemone *Nematostella vectensis* (BLASTP), and the best resulting homologous sequences were further used for localization predictions.

Sequences of differentially expressed transporter transcripts were in silico translated into protein sequences; those that were predicted to localize to the plasma membrane or to the host's lysosome/vacuole were further analyzed using InterPro as a diagnostic tool (Mitchell et al., 2019). InterPro, that is comprised of multiple and diverse databases, provides functional analysis of protein sequences by predicting the presence of domains and important sites.

## 3 RESULTS

### 3.1 RNAseq analysis of freshly isolated and cultured algal symbionts

A *Breviolum minutum* transcriptome was assembled from free‐living cultured algae and from algal cells freshly isolated from *Exaiptasia diaphana* utilizing three biological replicates from each life stage. We assume that the freshly isolated symbionts faithfully represent the transcriptomic state *in hospite*, and hence, from now on these will be referred to as *in hospite*. The resulting de novo assembled transcriptome comprises 58,619 open reading frame contigs and GC percentage of about 51. These numbers are compatible with the published draft genome of *B. minutum* (Shoguchi et al., 2013).

**Figure 1:**
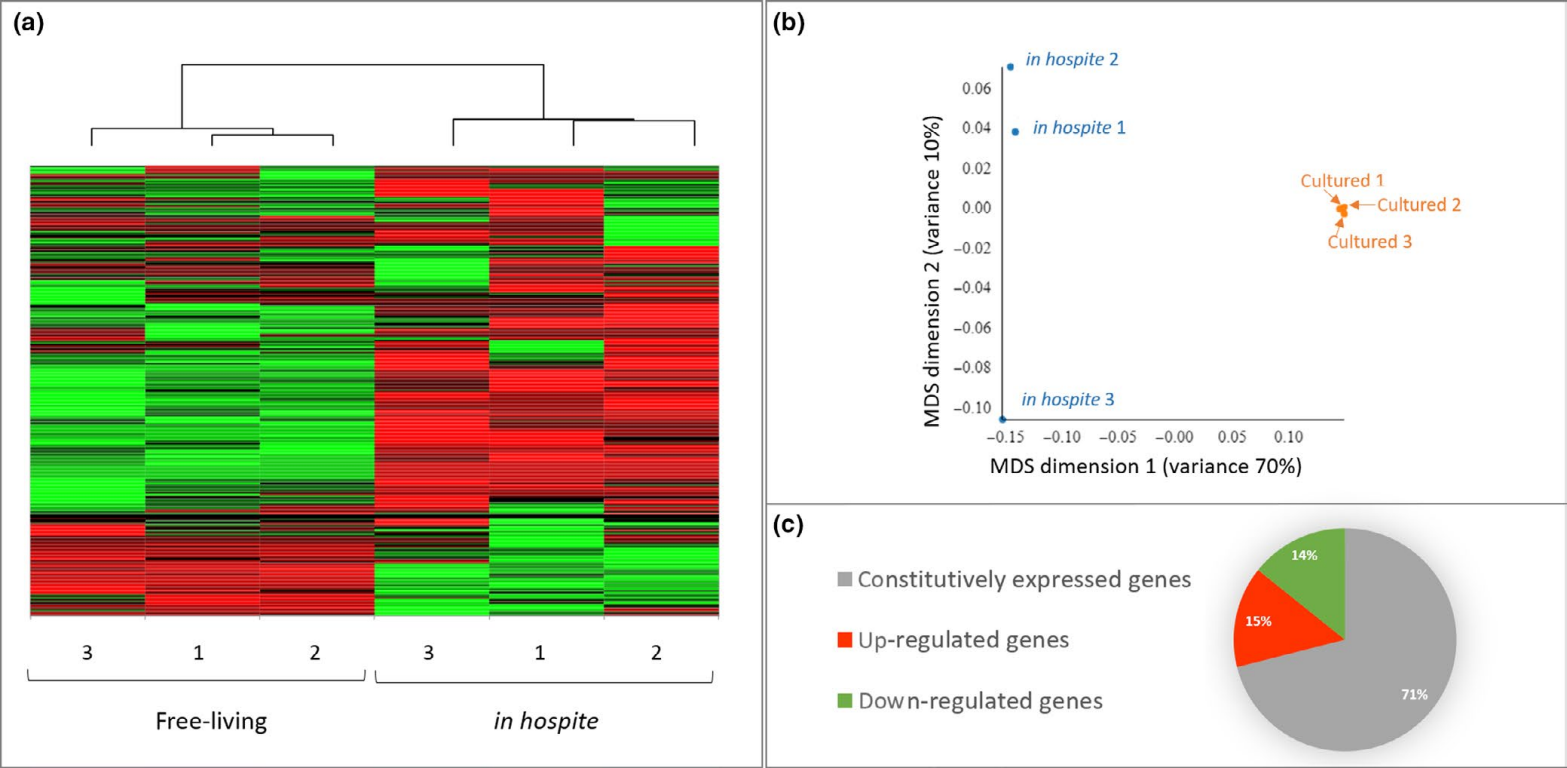
Clustering of free‐living cultured algal cells and *in hospite* samples. (a) Heat map of hierarchical clustering of all genes. Generated using TPM values (transcript per million) and based on Euclidian distances. (b) Multidimensional scaling (MDS) plot and percent of variance captured by the MDS dimensions. (c) Pie chart of percent of constitutively expressed, up‐ and down‐regulated genes in *in hospite* comparing to cultured *B. minutum*

Gene expression patterns of replicate samples from each life‐stage cluster more closely to one another than to samples of the other life stage (Figure 1a,b), with life stage explaining approximately 70% of the variance in the MDS dimension 1 (Figure 1b).

A total of 8,616 up‐regulated and 8,350 down‐regulated genes were identified for *in hospite* versus. cultured *B. minutum*, respectively. The 41,653 assembled transcripts, that were not significantly differentially expressed between *in hospite* and cultured algal cells, are henceforth referred to as “constitutively expressed” genes (Figure 1c).

**Figure 2:**
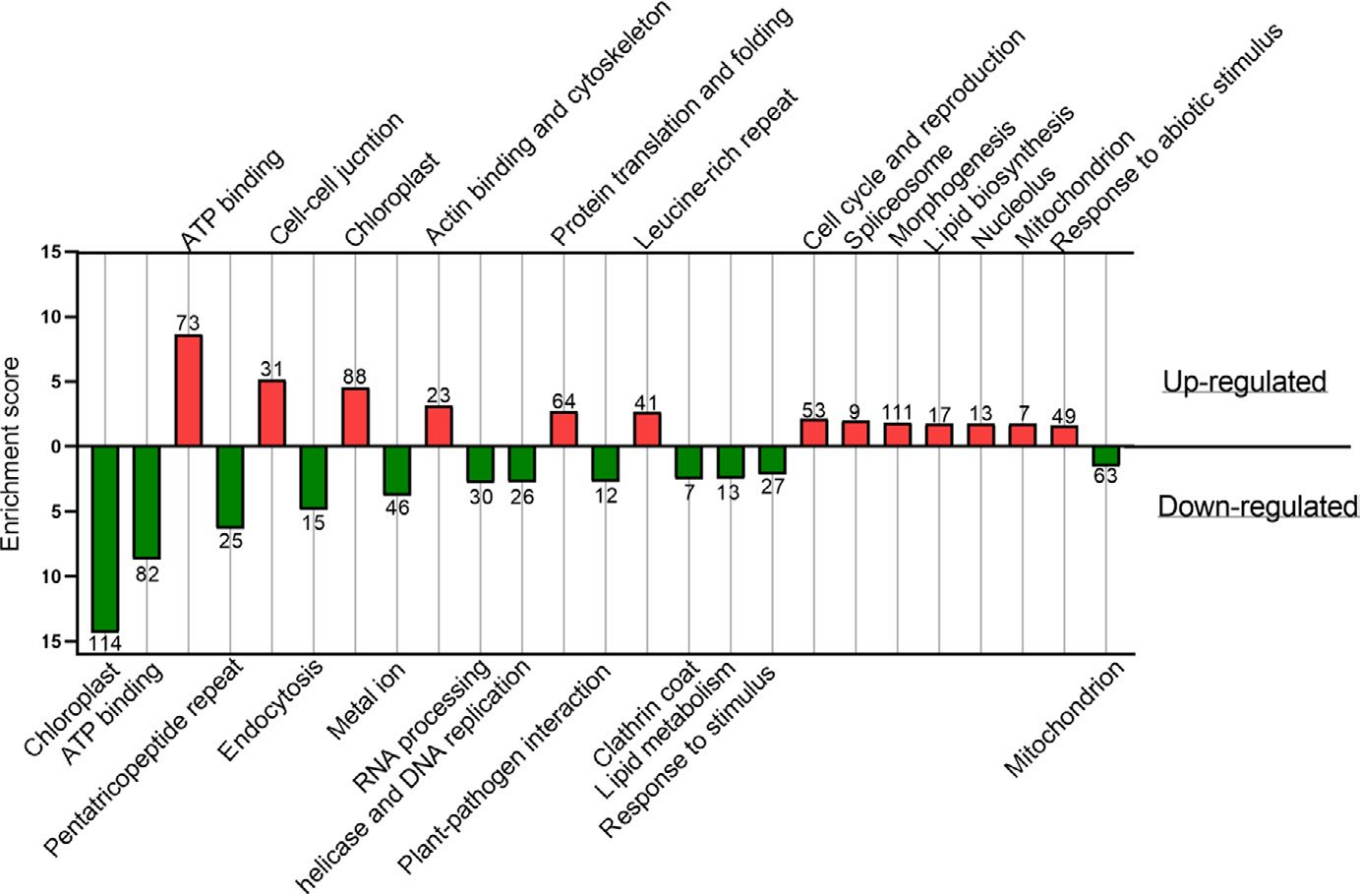
Enriched Gene Ontologies (GO) for differentially expressed genes of *in hospite* symbionts comparing to free‐living algae. Clusters of GO categories are presented for up‐regulated (red) and down‐regulated (green) genes and plotted against a calculated enrichment score. See Table S3 for details of the GOs categorizes within each group. The number of genes in each group is indicated on the column

**Figure 3:**
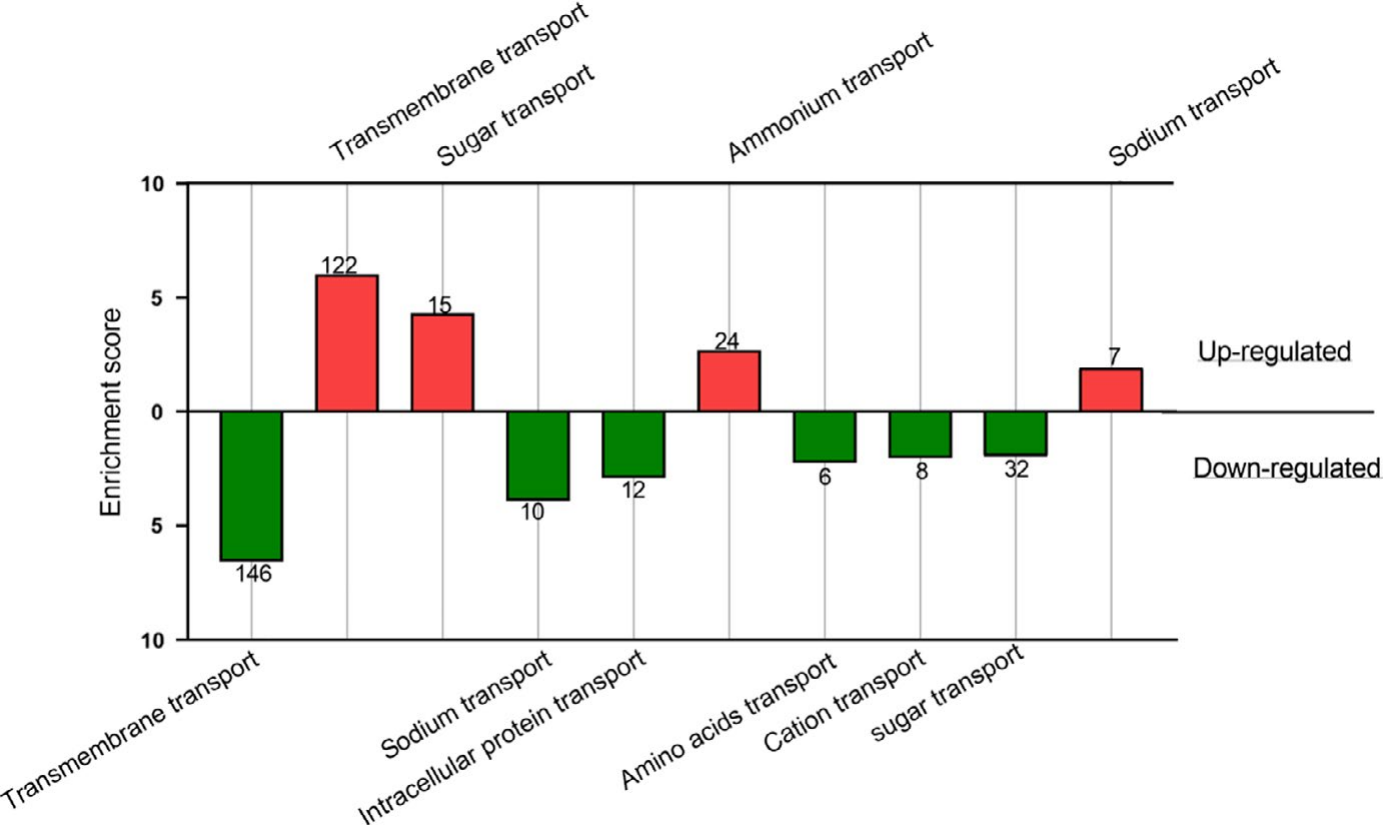
Enriched transport‐related Gene Ontologies (GO) for differentially expressed genes of *in hospite* symbionts comparing to free‐living algae. Clusters of GO categories are presented for up‐regulated (red) and down‐regulated (green) genes and plotted against a calculated enrichment score. See Table S3 for details of the GOs categorizes within each group. The number of genes in each group is indicated on the column

Enrichment analysis identified that ATP binding and chloroplast were the most enriched Gene Ontology (GO) groups in the case of down‐expressed and constitutively expressed genes but were also up‐regulated and high‐ranked for *in hospite* versus cultured symbionts (Figures 2 and S2; see Tables S3 and S4 detailing the GOs of every group). Processes related to protein translation and folding, spliceosome, and proteasome were up‐regulated, whereas RNA processing was down‐regulated. A large number of genes contributed to the up‐regulated group morphogenesis and development. Actin binding and cytoskeleton, cell cycle and reproduction, and leucine‐rich repeats, were also up‐regulated. Among the down‐regulated GOs, groups *in hospite* were endocytosis, pentatricopeptide repeat, metal ion, helicase and DNA replication, clathrin coat, and plant–pathogen interaction (Figure 2). Transmembrane transport was enriched in both up‐ and down‐regulated groups, with ammonium transport in the former and amino acid transport and cation transport in the latter group. Sugar transport was enriched, but with a twice higher score at the up‐regulated group, while sodium transport presented an opposite trend (Figure 3). Constitutively expressed genes were found to be significantly enriched for RNA processing, ubiquitin‐dependent protein catabolic process, response to heat, oxidation–reduction process, intracellular protein transport, endosome, protein kinase, among others (Figure S2, Table S4). A total of 213 up‐regulated and 167 down‐regulated potential “symbiosis transporters” were identified (Tables S5 and S6).

### 3.2 Differential expression of selected host transporter genes in aposymbiotic versus symbiotic anemones via qPCR

Eleven of the fourteen host candidate transporters of interest examined were found to be significantly down‐regulated in aposymbiotic anemones when compared to symbiotic anemones (Figure S1, Table S1): viz. ammonium transporter “rhesus‐like” (RH), ammonium transporter “AMT‐like,” calcium‐transporting ATPase, sodium‐dependent phosphate‐transport protein 2b, sodium‐dependent phosphate transporter 1, aromatic‐amino acid transporter 1, solute carrier family 22, organic cation transporter, monocarboxylate transporter 10, zinc transporter 1, and GABA and glycine transporter.

### 3.3 Putative localization of transporters of algal symbiont and host origin

**Figure 4:**
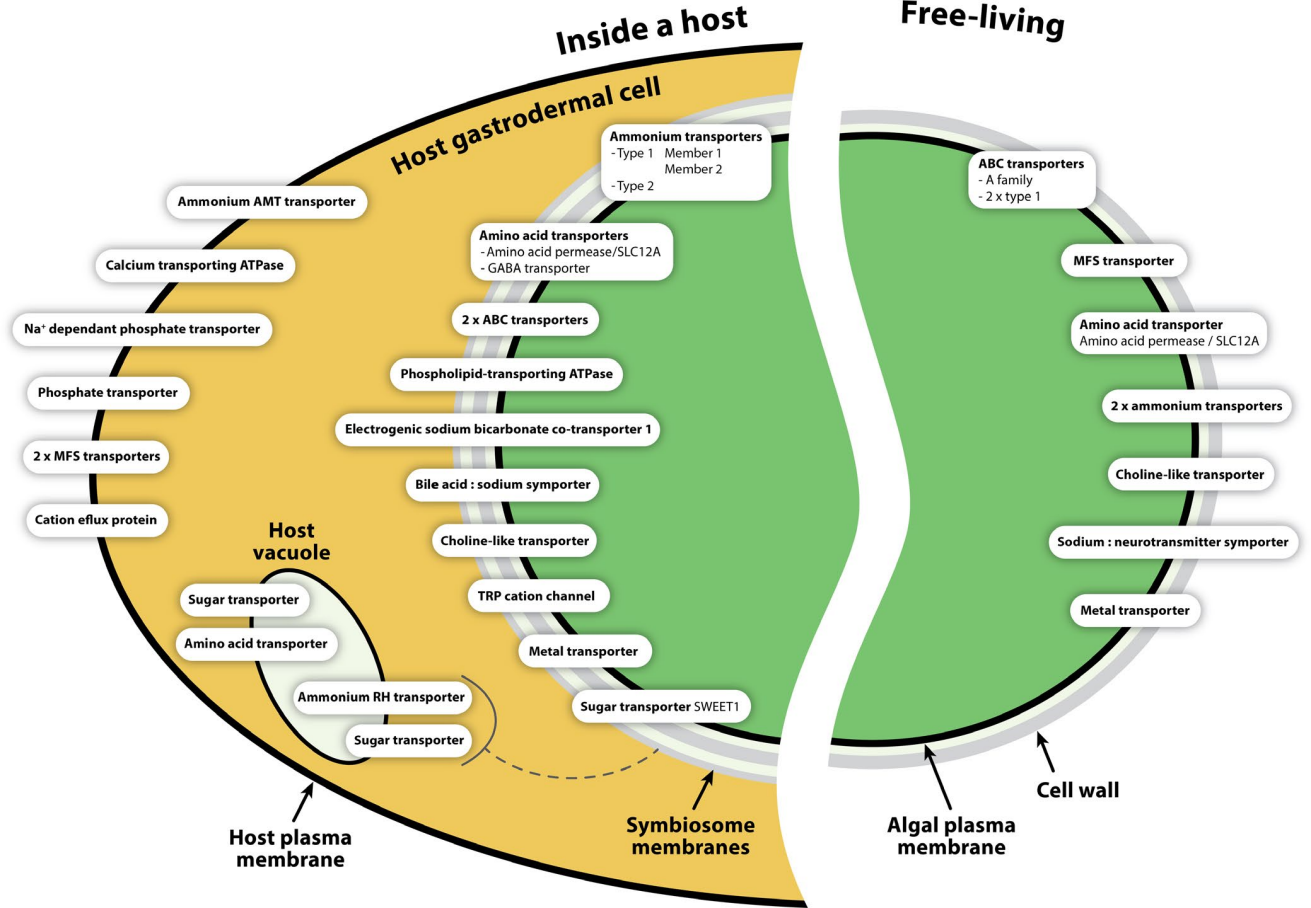
Illustration of differentially expressed transporters in the holobiont cell. Up‐ and down‐regulated transporters in the symbiont cell are presented along with up‐regulated host transporters. The transporter's label is indicative of its predicted domains or annotation. Subcellular location prediction (Almagro Armenteros et al., 2017) is demonstrated within the holobiont illustration. Two of the host's transporters that are predicted to the vacuole, are suspected to be localized on the symbiosome membrane, and are marked with a dashed line. See Tables S1, S5, and S6 for more details

Predicted locations for the up‐ and down‐regulated algal transporters place the transporters in six different subcellular compartments (Figure S3). Approximately 15% of the up‐regulated transporters and 17% of down‐regulated transporters were predicted to localize to the symbiont plasma membrane (Figure S3). Most of the 14 transporters of interest from the host anemones that we targeted were predicted to reside in the cell membrane; five had predicted localization in the lysosome/vacuole (see illustration in Figure 4).

An analysis targeted specifically at potential sugar transporters in the *B. minutum* transcriptome revealed that out of 134 transcripts (Table S7), only seven were predicted to be localized to the plasma membrane of the alga. Out of the putative plasma membrane transporters, just one transporter, SWEET1, is up‐regulated *in hospite*. Altogether, our transcriptome includes 10 SWEET transcripts of types: 1, 3, 6, 7, and 14.

## 4 DISCUSSION

### 4.1 Gene expression patterns of Symbiodiniaceae reflect *in hospite* and free‐living life stages

Certain members of family Symbiodiniaceae are known to exist both in a free‐living state (Coffroth, Lewis, Santos, & Weaver, 2006; Decelle et al., 2018) and as endosymbionts within cnidarian hosts (Stambler, 2011). Our transcriptome analysis on cultured and *in hospite Breviolum minutum* highlights how distinct the two life stages are, while the clustering of biological replicates is indicative of robustness in our analysis (Figure 1). However, we note that gene expression could potentially be affected by the extended handling during the symbiont isolation process prior to RNA extraction, and we cannot exclude that some portion of the variability between the free‐living and symbiotic algae is due to stress‐like response to the isolation procedures.

The environmental conditions of the cultured versus *in hospite* algal cell are highly distinct. Firstly, the cultures and the anemones were kept under different light intensities adjusted to their preferences. Moreover, the absorbance properties of the host tissue and self‐shading of symbiont cells within the host almost certainly results in even lower light availability for the symbionts (Stochaj & Grossman, 1997). Secondly, symbionts *in hospite* live within an osmotically different environment to that of the free‐living algae (Stambler, 2011). Thirdly, the space within the symbiosome is acidic (Rands, Douglas, Loughman, & Hawes, 1992; Venn et al., 2009), whereas in culture the cells are grown in seawater buffered to a pH of ~8.2, and the culture medium is heavily fortified with nutrients and vitamins. Thus, differential gene expression between cultured and endosymbiotic cells is likely to result from acclimatization or adaptation to highly different environmental conditions (Stochaj & Grossman, 1997).

Our findings show that for *B. minutum* living inside a host as opposed to living in culture triggers activation and down‐regulation of genes (Figure 2a). Correspondingly, GO categories related to protein translation and folding, spliceosome, and nucleolus were up‐regulated, while RNA processing was down‐regulated. Noteworthy, in a recent study *Durusdinium trenchii* was found to be highly enriched with mRNA splicing via spliceosome *in hospite* when compared to free‐living cultures of the same species (Bellantuono, Dougan, Granados‐Cifuentes, & Rodriguez‐Lanetty, 2018).

#### 4.1.1 Differentially expressed genes *in hospite*

Enrichment in the chloroplast GO category was expected since differences in photosynthesis (Bhagooli & Hidaka, 2003; Chen et al., 2012; Deane & O'Brien, 1978; Dubinsky et al., 1990; Stambler & Dubinsky, 2005) and in chloroplast content (Pasaribu et al., 2015) have been shown to occur between *in hospite* and cultured Symbiodiniaceae. However, in our analyses only one photosynthesis‐related term was enriched in the down‐regulated group and was not statistically significant (*p*val = .07). In addition to inorganic carbon fixation, the chloroplast provides multiple functions such as assimilation of ammonium, assembly of iron–sulfur complexes, and stress signaling (Dorrell & Howe, 2012), which are evident in the collection of differentially expressed genes in this chloroplast group. These are expected to be apparent in our RNAseq data since most (87%) of the plastid‐associated genes in *B. minutum* genome are encoded by the nuclear genome (Mungpakdee et al., 2014).

Gene expression changes in lipid biosynthesis and metabolism correspond with earlier findings showing that cellular lipid profiles are different in symbiotic versus cultured Symbiodiniaceae in the genus *Cladocopium* (Chen et al., 2015; Hambleton et al., 2019). Moreover, lipid droplets in the gastrodermal cells of the host were found to be dependent on the symbiotic state of the host (Luo et al., 2009). Since the symbiont cell is the primary site for lipid synthesis (Crossland, Barnes, & Borowitzka, 1980), it was suggested that most lipids are generated by the algal symbiont and sequentially transferred to the host cell in a format of lipid droplets (Peng et al., 2011). We postulate that phospholipid‐transporting ATPase, which is up‐regulated in *in hospite* and predicted to be localized to the symbiont cell membrane, might assist in this process. Additionally, the choline‐like transporters could be related to these lipid modifications, as they are known for transporting choline for phospholipid synthesis (Michel, Yuan, Ramsubir, & Bakovic, 2006). Symbiodiniaceae gene content was found to be enriched with choline transporters relatively to other eukaryotes (Aranda et al., 2016).

Enrichment of cell cycle and reproduction in the up‐regulated genes of the *in hospite* algae appears contrary to reduced symbiont growth rates *in hospite* (reviewed by Davy et al., 2012). A healthy animal host has a steady‐state algal density (Muscatine & Pool, 1979), and mitotic index is higher in culture (Davy et al., 2012; Wilkerson et al., 1988; Wilkerson, Muller‐Parker, & Muscatine, 1983). Thus, we would have expected that cell cycle and reproduction genes will be down‐regulated *in hospite*. Importantly, GO terms of helicase and DNA replication, which included genes of several DNA polymerase and DNA replication licensing factor, were in fact down‐regulated, which contradicts an apparent trend for faster replication in the cell cycle and reproduction GO categories.

#### 4.1.2 Up‐regulated genes *in hospite*

Upon entering a host cell, Symbiodiniaceae cells undergo metamorphosis from a flagellated motile stage to a coccoid stage (Trench, 1993), morphological and developmental processes that we expected to be evident in gene expression. A large number of morphogenesis‐related genes were indeed up‐regulated, along with cytoskeleton‐related genes. The symbiotic metamorphosis also encompasses changes in the cell wall: Within the host, the cell wall is thinner and structurally different compared with that of cultured cells (Palincsar et al., 1988; Pasaribu et al., 2015; Wakefield et al., 2000). Increased thickness of the granular layer of the Symbiodiniaceae cell wall in culture might be an adjustment to independent living outside of the host (Palincsar et al., 1988). We propose that gene expression changes in the GO categories for plasmodesmata, symplast, and cell–cell junction (cellular component), which are categories more attuned to multicellular plants where channels traverse the cell walls and enable communication between cells, represent changes in endosymbiotic cell wall in these unicellular algae (Williams, Lemoine, & Sauer, 2000). Overall, our list of genes exhibiting transcriptional controls linked to the symbiotic life stage likely underpins algal metamorphosis and provides a key to unraveling the genomic basis of Symbiodiniaceae/cnidarian symbiosis.

Leucine‐rich repeat (LRR) proteins in Toll‐like receptors are associated with the first line of defense in the innate immune response of cnidarians (Dunn, 2009). LRR domains typically comprise 2–45 motifs of 20–30 amino acids in length (Ng & Xavier, 2011) and provide a structural framework for protein–protein interactions (Kobe & Kajav, 2001). The draft genome of a *Cladocopium* sp. displays expansions of LRR (Shoguchi et al., 2018). Further, LRR‐kinase protein was previously identified in a proteomic analysis of Symbiodiniaceae cells freshly isolated from *Exaiptasia diaphana* (Peng et al., 2010; formerly *pulchella* [Grajales & Rodríguez, 2014]) and was differentially expressed compared with free‐living cultured cells (Pasaribu et al., 2015). However, since LRRs are known to be involved in a variety of biological processes, including cell adhesion, transcription, RNA processing, signal transduction, immune response, and DNA repair (Kobe & Kajav, 2001), we cannot infer at this stage what the biological significance of up‐regulation of this domain in the *in hospite* symbionts represents.

#### 4.1.3 Down‐regulated genes *in hospite*

Our analysis was largely focused on up‐regulated genes and their possible role in the alga–cnidarian symbiosis. However, enrichment analysis of down‐regulated genes revealed some noteworthy patterns. As a consequence of an alga being confined in a symbiosome, it is unlikely that particles are acquired, which is corroborated by the observed down‐regulation of the endocytosis pathway *in hospite*. Thus, we speculate that free‐living algae utilize endocytosis for the uptake of molecules that are too big for membranal protein‐assisted transport (Kalinina, Matantseva, Berdieva, & Skarlato, 2018), possibly as a form of heterotrophic feeding (Jeong et al., 2012; Xiang, Hambleton, Denofrio, Pringle, & Grossman, 2013; Zhang et al., 2014). Correspondingly, clathrin—which is the core of the clathrin‐dependent endocytosis pathway (Pearse, 1976)—is also down‐regulated *in hospite*. Additionally, confinement in a symbiosome might shelter symbionts from pathogen exposure, which might explain why genes in the plant–pathogen interaction pathway are down‐regulated *in hospite*.

RNA processing involves a range of RNA‐binding proteins, many of which contain pentatricopeptide repeats (PPR) that are defined by a 35‐amino acid structural motif (Fujii & Small, 2011). The down‐regulation of PRR proteins can be thus correlated with the down‐regulation of RNA processing in *B. minutum* *in hospite*. Almost all of the PPR proteins are targeted to plastids and mitochondria, where they function in post‐transcriptional processes including splicing and RNA editing (Cheng et al., 2016). PPR proteins were previously identified in *B. minutum* (Shoguchi et al., 2013) and in *Symbiodinium microadriaticum* (Liew, Li, Baumgarten, Voolstra, & Aranda, 2017) genomes in large numbers and were hypothesized to be related with RNA editing (Shoguchi et al., 2013) in response to environmental stress (Liew et al., 2017). Differential expression of PPR proteins was documented following exposure to thermal stress and phosphate depletion in *Fugacium kawagutii* (Senjie Lin, Yu, & Zhang, 2019) and following nitrogen supplement to nitrogen‐depleted cultures of the free‐living dinoflagellate *Karenia brevis *(Morey et al., 2011). If PPR are indeed related to environmental stress, their down‐regulation in our study might further support that the symbiosome is a stress‐reduced environment.

A cluster of 42 down‐regulated genes related to glycoproteins (N‐glycan biosynthesis and secretion) was identified, and while not significantly enriched, it might be related to host–symbiont recognition. It has been suggested that recognition at the initial phase of the symbiosis involves glycans that are on the surface of the algal cells that are identified by the cnidarian host (Kvennefors, Leggat, Hoegh‐Guldberg, Degnan, & Barnes, 2008; Kvennefors et al., 2010), in a similar manner to many other symbiotic systems (Gust, Willmann, Desaki, Grabherr, & Nürnberger, 2012; Nyholm, Deplancke, Gaskins, Apicella, & McFall‐Ngai, 2002). We assume that once inside a host those recognition systems are of reduced relevance and are thus down‐regulated.

#### 4.1.4 Previous omics studies that compared *in hospite* and cultured Symbiodiniaceae

Two very recent studies describe transcriptional changes in *in hospite* symbionts versus. the free‐living stage in other Symbiodiniaceae species. Transcriptional changes were described in the algal symbiont *Cladocopium goreaui* after infecting planula larvae of the coral *Acropora tenuis* (Mohamed et al., 2019). The transcriptomic response of *C. goreaui* to the symbiotic state was complex, and the most obvious feature was extensive suppression of gene expression that included down‐regulation of genes related to protein synthesis, N‐glycan biosynthesis, and a range of stress response and immune‐related genes. In contrast, genes implicated in metabolism were up‐regulated in the symbiotic state. The transcriptomic response of *C. goreaui* to symbiosis implied that the mutualistic relationship, including translocation of metabolites, can be established already at the larval stage (Mohamed et al., 2019). Bellantuono et al. (2018) compared the transcriptomes of *Durusdinium trenchii in hospite* versus. free‐living, under ambient conditions and thermal stress. Similar to *in hospite B. minutum*, *in hospite D. trenchii* exhibited a distinct gene expression pattern when compared to its cultured counterparts. Under stable conditions, *in hospite D. trenchii* demonstrated lower transcriptional activity than the cultured algae, suggesting that the host provides a stabilized environment for its algal symbionts. However, the opposite trend was documented when *in hospite D. trenchii* was subjected to thermal stress, indicating an exacerbated stress environment within the host cell (Bellantuono et al., 2018).

Two previous studies focused on proteome differences of the two life stages and identified a few key proteins using immunoblotting (Stochaj & Grossman, 1997) or mass spectrometry (Pasaribu et al., 2015). Stochaj & Grossman, 1997, used one‐ and two‐dimensional protein gels to visualize the differences in four major polypeptides, which they characterized and identified as peripheral membrane peridinin–chlorophyll, the integral membrane peridinin–chlorophyll *a*/*c*‐binding proteins, and the large subunit of RuBP carboxylase (Stochaj & Grossman, 1997). In our data, some transcripts of the latter two were significantly up‐regulated, while the former were not identified (Table S8). Mass spectrometric analysis of freshly isolated Symbiodiniaceae cells identified twelve distinct proteins that included transcription translation factors, photosystem proteins, and proteins associated with energy and lipid metabolism as well as defense response candidates (Pasaribu et al., 2015). Even though some of these trends correlate with our analysis (Acot13 of lipid metabolism and LRR kinase; Table S8), others were not apparent in our transcriptome. Other studies of expression differences of *in hospite* versus free‐living Symbiodiniaceae focused on the p‐type H^+^‐ATPase transporter and its possible role in carbon supply mechanism (Bertucci, Tambutté, Tambutté, Allemand, & Zoccola, 2009; Mies et al., 2017). This transporter was not present in our transcriptome data. Those inconsistencies are perhaps attributable to the high throughput properties of RNA sequencing (McGettigan, 2013), or poor correlation between mRNA quantities and steady‐state amounts of a proteins (Sealfon & Chu, 2011), or simply reflect different experimental set ups, or even the different species being studied (Bertucci et al., 2009).

### 4.2 Candidate “symbiosis membranal transporters”

Since transmembrane transport was highly enriched in both the down‐ and up‐regulated groups, transport clearly holds great significance to the symbiotic relationship, and we decided to further focus on membranal transporters in our analysis. Using bioinformatic prediction tools, we characterized differentially expressed transporters (Figures 3 and 4), assuming that those that are up‐regulated *in hospite* might have a prominent role in the symbiotic exchange of metabolites. Conversely, down‐regulated transporters may highlight key features of the free‐living state and the capacity of these rapidly growing algae to acquire nutrients from their media. However, we would like to stress the shortcoming of bioinformatic prediction as only experimental demonstration can determine with more certainty the subcellular location of a protein.

#### 4.2.1 Algal sugar transporters

We expected that sugar transport will be increased *in hospite* to satisfy the increased demand for organic sugars from the cnidarian host. However, “sugar transport” GO categories were documented in both up‐ and down‐regulated groups as well as constitutively expressed genes, which paints a more complicated picture. Nevertheless, sugar transport was twofold enriched (enrichment score) in the up‐regulated group, compared with the down‐regulated group. Moreover, an examination of the list of potential sugar transporters (Table S7) revealed 36 up‐regulated and only 13 down‐regulated genes.

Notably, 64% of the putative sugar transporters identified in our transcriptome were not differentially expressed. This corresponds with a study that documented relatively small changes in the expression of glucose transporters after supplementing *B. minutum* cultures with glucose, which concluded that these sugar transporters are not transcriptionally regulated (Xiang et al., 2018) but likely controlled at the post‐transcriptional level, either by RNA editing (Liew et al., 2017) or by microRNA (Aranda et al., 2013; S. Lin et al., 2015).

Two bicarbonate transporter isoforms predicted to be located on the plasma membrane of the symbiont are up‐regulated *in hospite* (Figure 4), possibly in order to provide inorganic carbon (Furla, Allemand, & Orsenigo, 2000; Zoccola et al., 2015) for the growing need for raw material for photosynthesis while *in hospite*.

The sole up‐regulated sugar transporter predicted to be localized to the symbiont plasma membrane belongs to the recently identified novel family of SWEET (SWEET1) transporters (Chen et al., 2010). SWEETs were first identified in plants (Chen et al., 2010), but homologs exist in all kingdoms of life (Feng & Frommer, 2015). In plants, they are responsible for efflux and intracellular trafficking of sugars (Deng & Yan, 2016) and are localized to different cellular compartments (Feng & Frommer, 2015). SWEETs were previously identified in a *B. minutum* transcriptome (strain SSB01), and three out of seven transcripts were up‐regulated when the medium was supplemented with glucose (Xiang et al., 2018). Intriguingly, all but one (which did not pass the prediction probability threshold) of the SWEET transcripts in our transcriptome are predicted to reside in the plasma membrane. Thus, SWEET transporters may have a key role in transporting sugars into and/or out of *B. minutum* cells, and SWEET1 may be used by the symbiont to feed its host.

#### 4.2.2 Algal nitrogen and phosphate transporters

Our data further highlight the importance to nitrogen cycling in the algal–cnidarian relationship (Rädecker, Pogoreutz, Voolstra, Wiedenmann, & Wild, 2015; Rädecker et al., 2018). We observed up‐regulation of symbiont and host ammonium transporters in symbiosis. Up‐regulation of ammonium transporters and assimilation was also found in *in hospite Durusdinium trenchii* when compared with free‐living algal symbionts (Bellantuono et al., 2018). Nitrogen is essential for normal cell maintenance and particularly for production of amino acids. In the cnidarian holobiont, nitrogen is at the crux of the resources limitation theory, which posits that the host limits the supply of nitrogen to the algal symbiont to control its numbers (Davy et al., 2012).

In cnidarian symbioses, either nitrate or ammonium can serve as nitrogen sources (Yellowlees, Rees, & Leggat, 2008), though ammonium is favored (Grover, Maguer, Reynaud‐Vaganay, & Ferrier‐Pagès, 2002). Both the host and the alga can assimilate ammonium, but it seems that the alga is the primary site for this assimilation (Pernice et al., 2012). Overall, the endosymbionts are heavily dependent upon host‐derived recycled nitrogen, in addition to nitrogen derived from seawater (Tanaka, Grottoli, Matsui, Suzuki, & Sakai, 2015). Fixation of ammonium into amino acids by the host likely facilitates ammonium transport into the endosymbiont by creating a favorable concentration gradient (D'Elia, Domotor, & Webb, 1983; Pernice et al., 2012). Our results showing up‐regulation of ammonium transporters, combined with putative localization to the symbiont plasma membrane, support the hypothesis that the endosymbiont acts as an “ammonium sink” within the holobiont (Aranda et al., 2016).

Glutamine synthetase and glutamine 2‐oxoglutarate amido transferase (GS/GOGAT pathway) underpin symbiont ammonium assimilation (Davy et al., 2012). Our transcriptome only identified glutamine synthase, but with no obvious trend (some of the identified transcripts were up‐regulated, while others were down‐regulated or not differentially expressed at all; Table S9). Conversion of nitrate to ammonium is achieved exclusively by the symbiont using nitrate and nitrite reductases (Davy et al., 2012). Four out of seven transcripts of nitrate reductase, and three nitrite reductase transcripts, are up‐regulated *in hospite*. The ammonium generated by these reductases is assimilated into amino acids, with essential amino acids being translocated to the host (Wang & Douglas, 1999). Candidates for this essential amino acid translocation are the amino acid permease and/or the GABA transporter, both of which we show to be up‐regulated *in hospite* and putatively localized to the symbiont plasma membrane. Essential amino acids are defined as those that cannot be synthesized by an organism. However, there is no consensus on whether amino acids considered to be essential in most animals are also essential for cnidarians (Wang & Douglas, 1999). De novo synthesized amino acids will also be used by the symbiont itself (Pernice et al., 2012) or be temporarily stored as a compound with high nitrogen content, such as uric acid crystals (Clode, Saunders, Maker, Ludwig, & Atkins, 2009). Dinoflagellates can also take up amino acids from seawater (Kopp et al., 2013), which might explain why amino acids transporters are mostly down‐regulated *in hospite*, and there being a greater need for amino acid scavenging in the free‐living state.

#### 4.2.3 Host transporters

All molecules transferred between the host and symbiont must cross through the symbiosome membrane. Two *Nematostella vectensis* sequences homologous to host transporters (ammonium “rhesus‐like” [RH] transporter and a sugar transporter [Table S1]) differed in their predicted localization from in *E. diaphana*: In *E. diaphana,* these were predicted to be localized to the lysosome/vacuole, but in *N. vectensis* the transporters are predicted to be in the plasma membrane. This might suggest their localization to the *E. diaphana* symbiosome (Sproles et al., 2018; Figure 4). Thus, those transporters are attractive candidates for transporting the essential compounds ammonium and sugar across this interface.

Nitrogen cycling has been shown to be drastically different between symbiotic and aposymbiotic cnidarians (Rädecker et al., 2018). Algal photoautotrophy requires transport of inorganic carbon, nitrogen, phosphate, and other inorganic nutrients through the host tissues (Yellowlees et al., 2008). Thus, a symbiotic cnidarian host will need to satisfy a greater need for inorganic nutrients than an aposymbiotic cnidarian. This requirement is apparently manifested in up‐regulation in symbiotic anemones of the ammonium AMT transporter predicted to localize to the host plasma membrane in our analysis.

Similarly, up‐regulation of two putative host plasma membrane phosphate transporters during symbiosis is congruent with greater demands for inorganic nutrients by the holobiont. Since aposymbiotic corals (Muller‐Parker, Cook, & D'Elia, 1990) are unable to take up phosphate from seawater (Davy et al., 2012), and the symbiont is proposed to act as a sink for phosphate within the holobiont (Godinot, Ferrier‐Pagés, & Grover, 2009). However, relatively little is known about phosphate acquisition in the alga–cnidarian symbiosis (Yellowlees et al., 2008), and further research is needed to better understand phosphate cycling in the holobiont.

#### 4.2.4 Other algal transporters

Na^+^ is a commonly cotransported ion in “secondary transporters,” which utilize the electrochemical gradient to translocate a second ion or a molecule against its gradient across membranes (Krishnamurthy, Piscitelli, & Gouaux, 2009). For this reason, the enrichment in Na^+^ transport documented here is difficult to allocate to any process in the algal cell at this stage.

Transporters with ATP binding cassette (ABC) domains and putative localization within symbiont cell membrane were both up‐ and down‐regulated. Recently, transcripts of an ABC transporter were found to be up‐regulated in another Symbiodiniaceae species, *D. trenchii*, when *in hospite* when compared with free‐living algal cells (Bellantuono et al., 2018). ABC transporters comprise a large family of transport proteins powered by ATP hydrolysis. In eukaryotes, ABC transporters are exporters (bind substrates from the cytoplasm and transfer them across a membrane) that transport a variety of substrates such as ions, sugars, amino acids, and lipids (Coll & Tieleman, 2011). Thus, it is not feasible to speculate about the possible roles of algal ABC transporters *in hospite* or the free‐living state. Nevertheless, it is clear that transcription of this large category of algal transporters is responding to life‐stage changes.

## 5 CONCLUSIONS

This study reveals extensive changes in gene expression for the alga *B. minutum* depending on whether it is free‐living or in symbiosis with its anemone host. Gene expression changes correlate with both morphological and metabolic changes adopted during the two radically different life stages. Many substrate transporters, both in the symbiont and the host, are shown to undergo extensive gene expression changes as part of the symbiosis. Further, we pinpoint candidate transporters localized throughout the holobiont compartments that might serve pivotal roles in the interchange of materials during symbiosis. Transfer of sugar and ammonium between the symbiont and the host comprises the most significant exchange of compounds in the symbiosis, and we identify promising candidate transporters that may assist in these processes, which are putatively localized to the algal plasma membrane and the symbiosome membrane. Future studies should focus on clarifying the missing details of how the two organisms successfully interact as a holobiont, by confirming both the subcellular localization of these putative transporters and defining their actual substrate preferences. These insights will expand our understanding of the material exchanges that are the crux of the symbiotic relationship.

## CONFLICT OF INTEREST

None declared.

## AUTHOR CONTRIBUTIONS

KM‐L, MJHvO and GIM designed research. KM‐L performed research and analyzed data. MJHvO and GIM analyzed data. KM‐L, MJHvO and GIM wrote the paper.

## Data Availability

The Fastq files were deposited at SRA database under the accession number PRJNA544863.
