# Supplementary material for: Symbiotic lifestyle triggers drastic changes in the gene expression of the algal endosymbiont Breviolum minutum (Symbiodiniaceae)
Source: Ecol Evol. 2019 Dec 12;10(1):451–66. doi: 10.1002/ece3.5910 (PMC6972872; doi:10.1002/ece3.5910)
Supplement: Supplementary file 1 [file ECE3-10-451-s001.pdf]

SI

Symbiotic lifestyle triggers drastic changes in the gene expression of the algal endosymbiont *Breviolum minutum* (Symbiodiniaceae)

Keren Maor-Landaw[1]*, Madeleine J. H. van Oppen[1,2], Geoffrey I. McFadden[1]

[1]School of BioSciences, The University of Melbourne, Parkville, Melbourne, VIC 3010, Australia

[2]Australian Institute of Marine Science, PMB #3, Townsville, QLD 4810, Australia

*Corresponding author: Keren Maor-Landaw; Keren.Maor@live.com

Key words: symbiosis, transporters, *Breviolum minutum*, *Exaiptasia pallida*, free-living, *in hospite,* RNAseq, life stage

Running title: gene expression changes cnidarian host-algal symbiosis

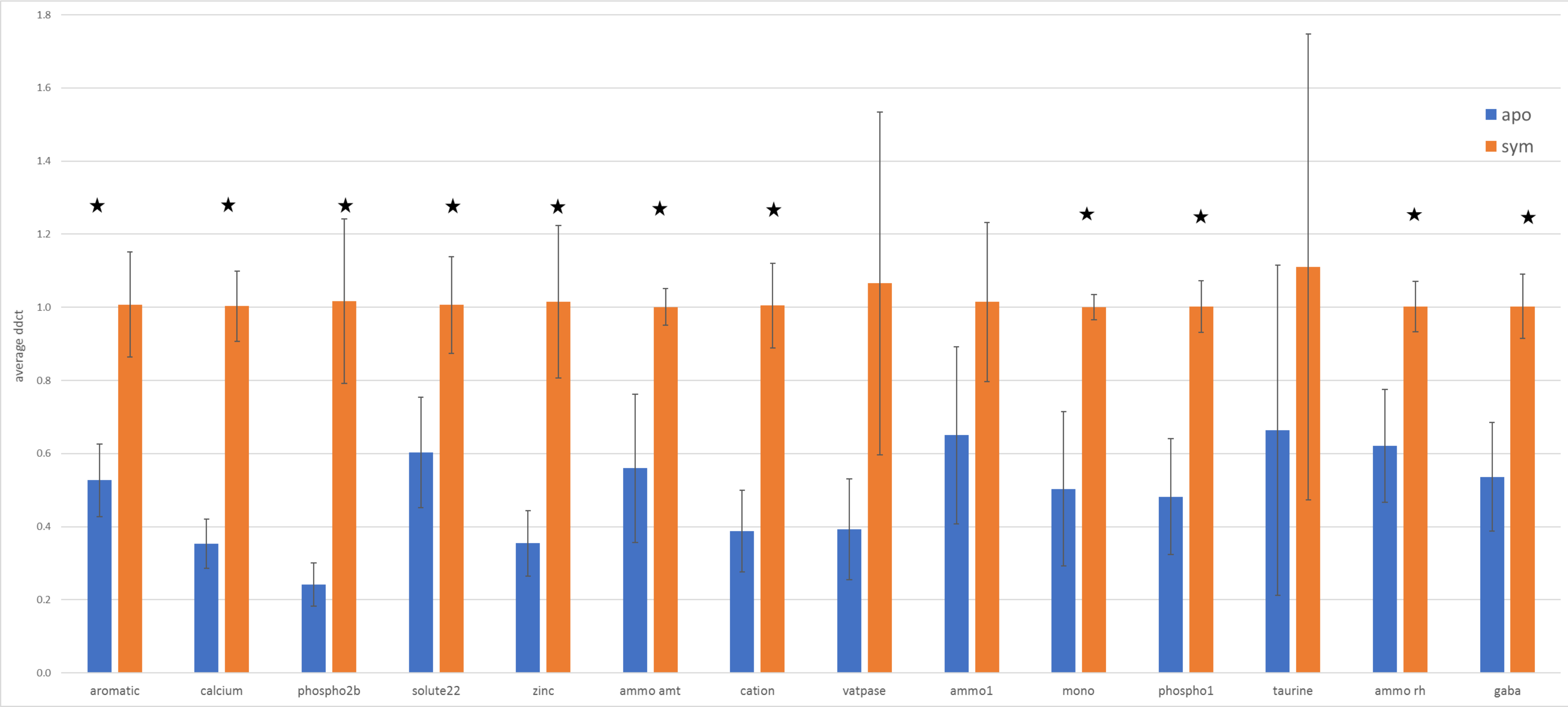

Figure S1 – Gene expression of host's candidate transporters-of-interest. Candidates retrieved from previous apo- vs. symbiotic host comparative papers (Lehnert et al., 2014; Oakley et al., 2016; Wolfowicz et al., 2016) were studied using Real Time PCR. Average ddct values of three *Exaiptasia pallida* genotypes of each apo- and symbiotic anemones, are presented. Significant (independent samples test (2-tail $p < 0.05$)) differences between apo- and symbiotic samples are indicated with a star. See table S1 for full annotation names and analysis of domain predictions. Apo; apo-symbiotic anemone, sym; symbiotic anemone.

Constitutively expressed algal genes

Enrichment analysis of constitutively expressed genes emphasized some processes that are not dependent upon the symbiotic state and can be categorized as house-keeping tasks (Fig. S2). However, some processes are common between the differentially and constitutively expressed, which points out that the expression of some genes under the same GO category was modified, while the expression of others was not.

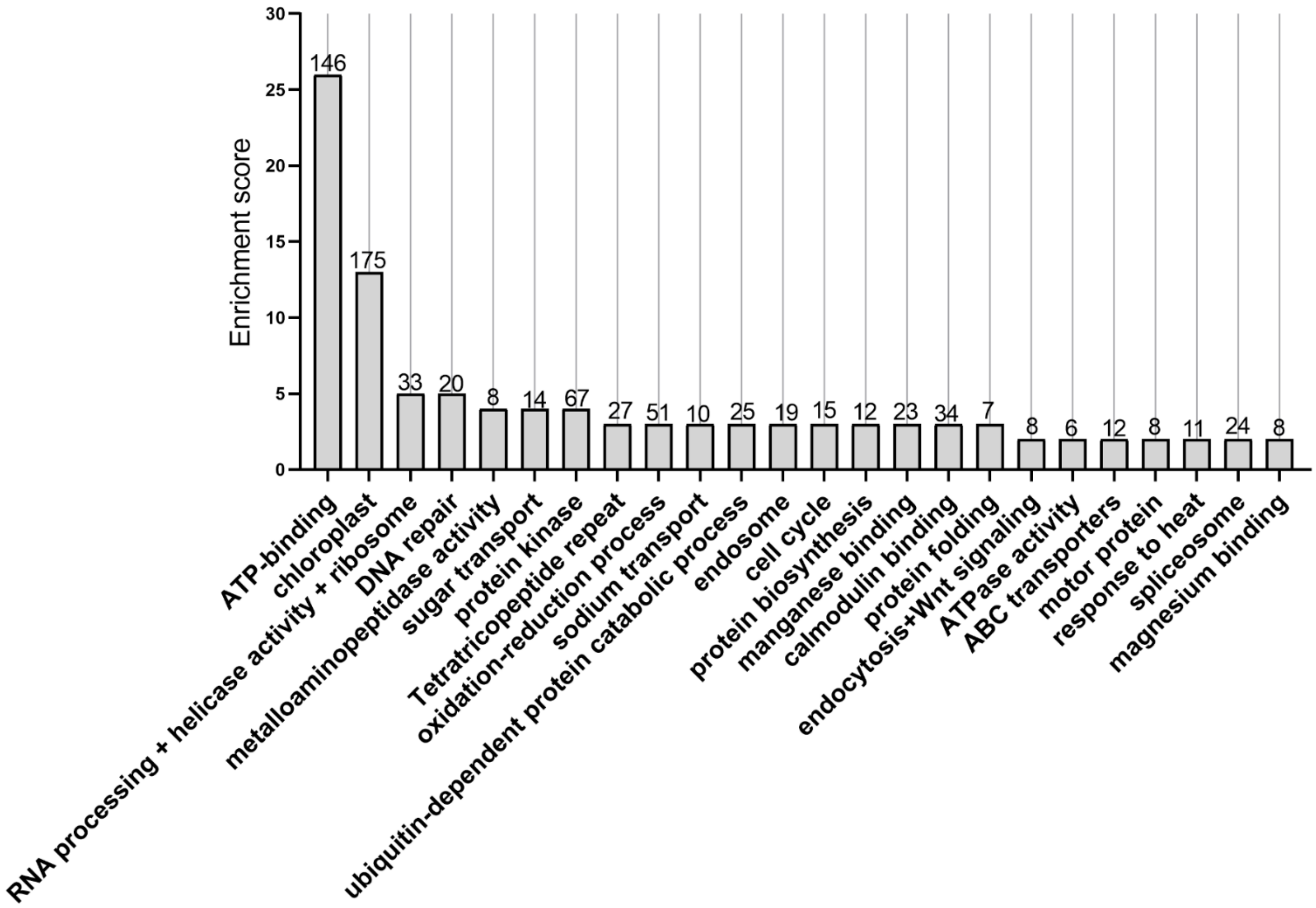

Figure S2 - Enrichment score of Gene Ontologies groups of ‘constitutively expressed’ genes (are not significantly differentially expressed between *in hospite* and cultured). See Table S4 for details of the GOs categorizes within each group. The number of genes in each group is indicated.

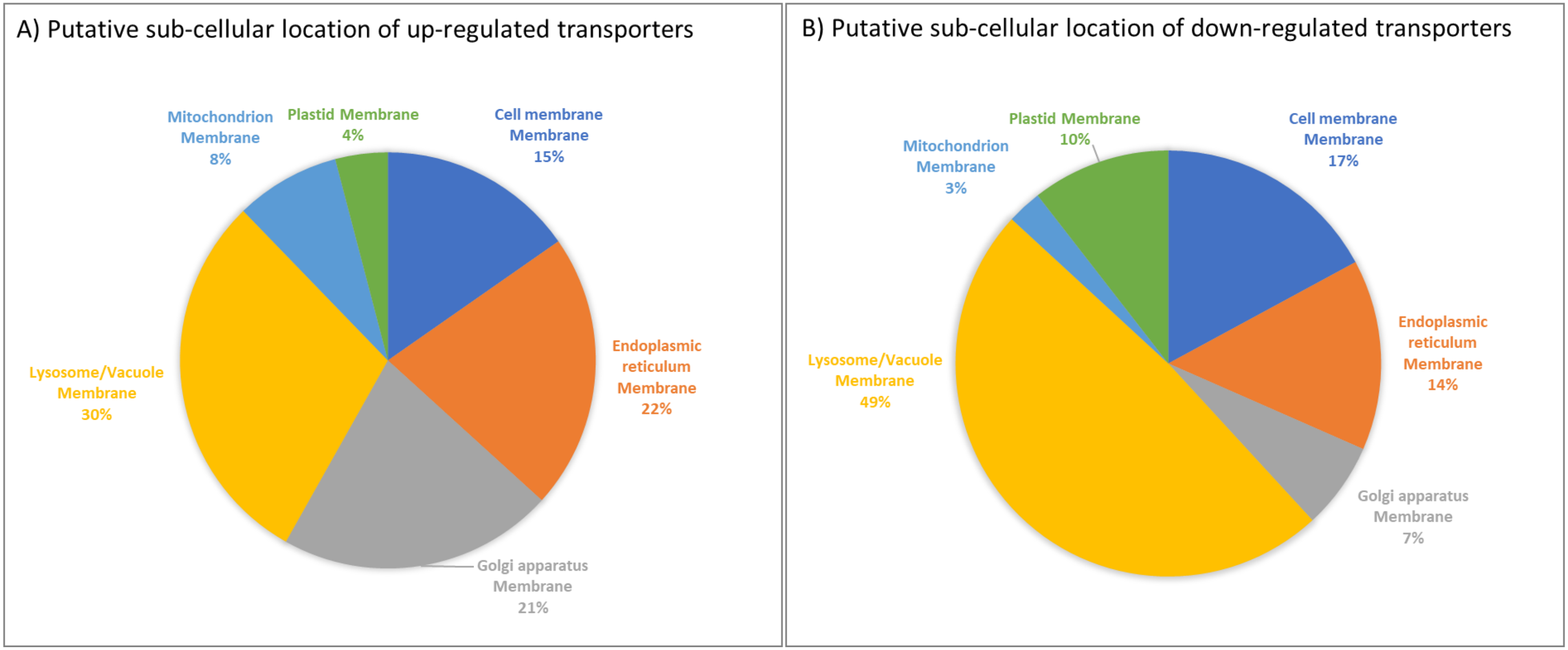

Figure S3 – Predicted sub-cellular location of up- (A) and down-regulated (B) candidate transporters. Prediction was conducted using DeepLoc-1.0 (Almagro Armenteros et al., 2017). Data is shown only for proteins that were predicted to be membranal and were ranked with high a hierarchical tree likelihood (>0.5).
